# Adjunct Terbinafine in Patients With Leukemia and Invasive Fusariosis With Skin Lesions: Discordance Between Responses of Skin Lesions and Systemic Outcomes

**DOI:** 10.1093/ofid/ofae068

**Published:** 2024-02-13

**Authors:** Takahiro Matsuo, Sebastian Wurster, Ying Jiang, Jeffrey Tarrand, Dimitrios P Kontoyiannis

**Affiliations:** Department of Infectious Diseases, Infection Control and Employee Health, The University of Texas MD Anderson Cancer Center, Houston, Texas, USA; Department of Infectious Diseases, Infection Control and Employee Health, The University of Texas MD Anderson Cancer Center, Houston, Texas, USA; Department of Infectious Diseases, Infection Control and Employee Health, The University of Texas MD Anderson Cancer Center, Houston, Texas, USA; Section of Clinical Microbiology and Virology, Division of Pathology and Laboratory Medicine, The University of Texas MD Anderson Cancer Center, Houston, Texas, USA; Department of Infectious Diseases, Infection Control and Employee Health, The University of Texas MD Anderson Cancer Center, Houston, Texas, USA

## Abstract

**Figure:**
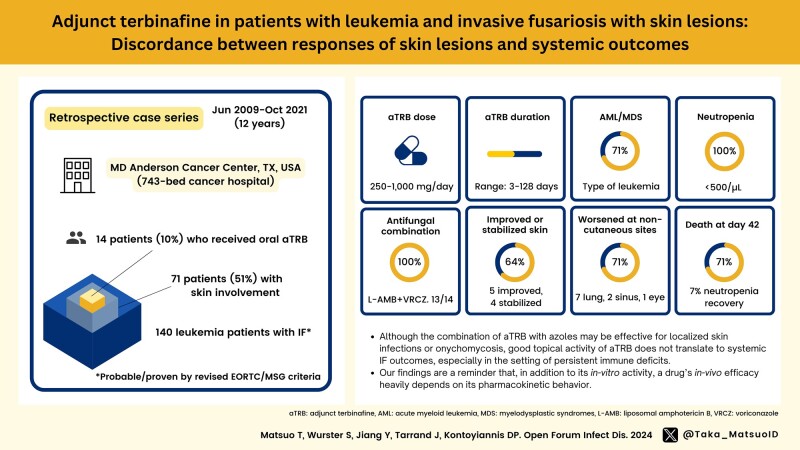

To the Editor—Invasive fusariosis (IF) is a severe opportunistic mold infection that often results in poor outcomes, especially for patients with hematological malignancies [1–4]. Skin involvement is the second most common manifestation of IF after pulmonary involvement [1–3]. While in vitro studies showed synergistic effects of the squalene epoxidase inhibitor terbinafine (TRB) when combined with triazoles [5] or liposomal amphotericin B (L-Amb) [6] against various fungal pathogens, including *Fusarium* species, clinical data to support these findings are limited. As TRB has poor penetration in lungs and other visceral tissues, in contrast to its excellent concentration in skin [7], we hypothesized that there might be discordance between good responses of *Fusarium* skin lesions to TRB but poor systemic outcomes in IF patients treated with adjunct TRB (aTRB). Therefore, we retrospectively reviewed adult leukemia patients (aged ≥18 years) with culture-documented IF at MD Anderson Cancer Center (Houston, Texas) from 2009 to 2021. Among 140 IF patients (114 proven, 26 probable, European Organization for Research and Treatment of Cancer Mycoses Study Group definitions) [8], 71 patients (51%) had skin involvement. Of those, we identified 14 patients who received oral aTRB (dose range, 250–1000 mg/day; duration, 3–128 days) (Table 1). Their median age was 46 years (range, 21–80 years); 13 (93%) were male. Ten patients (71%) had acute myeloid leukemia/myelodysplastic syndrome. All patients had neutropenia (<500/µL) and 4 (29%) had undergone prior allogenic hematopoietic stem cell transplantation. The majority (13 patients [93%]) had disseminated skin lesions. In addition to aTRB, 13 patients received voriconazole + L-Amb and 1 received posaconazole + L-Amb. The median time from IF diagnosis to aTRB initiation was 8 days (range, 0–74 days). While skin lesions on day 42 after aTRB initiation or at the time of earlier death were improved (5 patients [36%]) or stabilized (4 patients [29%]) in 64% of patients, 10 of 14 (71%) had progression of IF in sites with poor pharmacologic exposures of aTRB (lungs in 7 patients, sinus in 2 patients, endophthalmitis in 1 patient). Notably, 71% of patients died within 42 days of aTRB initiation.

**Table 1.** Clinical Characteristics, Treatment, and Outcomes of 14 Patients With Hematologic Cancer and Invasive Fusariosis

Case	Age (y)/Sex	Malignancy, Status	Allo-HSCT	Site(s) of Infection	Blood Culture Positivity	Systemic Therapy	TRB Dose	TRB Duration, d	Outcome of Skin Lesions	IF Progression at Noncutaneous Sites	Died by Day 42	Neutrophil Recovery at Day 42^a^
1	21/F	ALL, active	No	Lung, skin	Yes	L-AmB + VRC + TRB	Oral 250 mg q8h	31	Worsened	Yes (lung)	Yes	No
2	37/M	CML, active	No	Lung, skin	No	L-AmB + VRC + TRB	Oral 250 mg q12h	17	Worsened	Yes (lung)	Yes	No
3	59/M	MDS, active	No	Lung, skin	No	L-AmB + POS + TRB	Oral 250 mg q12h	64	Improved	Yes (lung)	No	No
4	28/M	AML, active	No	Sinus, lung, skin	No	L-AmB + VRC + TRB	Oral 250 mg q12h	6	Worsened	Yes (sinus)	Yes	No
5	24/M	AML, active	Yes	Sinus, lung, skin	Yes	L-AmB + VRC + TRB	Oral 500 mg q12h	8	Worsened	Yes (sinus)	Yes	No
6	60/M	AML, active	No	Sinus, lung, skin	Yes	L-AmB + VRC + TRB	Oral 250 mg q8h	15	Stable	Yes (lung)	Yes	No
7	70/M	AML, active	No	Skin	No	L-AmB + VRC + TRB	Oral 250 mg q24h	8	Stable	No	Yes	No
8	29/M	AML, active	Yes	Lung, skin	Yes	L-AmB + VRC + TRB	Oral 250 mg q24h	5	Stable	Yes (lung)	Yes	No
9	41/M	MDS, active	No	Lung, skin	No	L-AmB + VRC + TRB	Oral 500 mg q12h	128	Stable	No	No	No
10	68/M	CML, active	No	Sinus, lung, skin	Yes	L-AmB + VRC + TRB	Oral 250 mg q24h	40	Improved	Yes (end-ophthalmitis)	Yes	No
11	58/M	AML, active	No	Lung, skin	No	L-AmB + VRC + TRB	Oral 250 mg q12h	9	Improved	No	Yes	No
12	31/M	AML, active	Yes	Sinus, skin	Yes	L-AmB + VRC + TRB	Oral 250 mg q12h	59	Improved	Yes (lung)	No	No
13	80/M	AML, active	Yes	Lung, skin	No	L-AmB + VRC + TRB	Oral 250 mg q12h	7	Improved	No	No	Yes
14	50/M	MF, active	No	Lung, skin	No	L-AmB + VRC + TRB	Oral 250 mg q12h	3	Worsened	Yes (lung)	Yes	No

Abbreviation: Allo-HSCT, allogenic hematopoietic stem cell transplant; ALL, acute lymphoblastic leukemia; AML, acute myeloid leukemia; CML, chronic myeloid leukemia; F, female; IF, invasive fusariosis; L-AmB, liposomal amphotericin B; M, male; MDS, myelodysplastic syndrome; MF, myelofibrosis; POS, posaconazole; q8h, every 8 hours; q12h, every 12 hours; q24h, every 24 hours; TRB, terbinafine; VRC, voriconazole.

^a^All patients were neutropenic (absolute neutrophil count <500 cells/µL) at the time of IF diagnosis.

Oral TRB is subject to rapid hepatic first-pass metabolism and has high lipophilicity, resulting in >90% steady-state distribution to skin and adipose tissue [7]. These pharmacokinetic limitations could be a plausible explanation for our findings. TRB’s robust bioavailability in skin and nails possibly underpins the observed improvement or stabilization of skin lesions. However, its poor tissue penetration in lungs and other systemic sites prevents its efficacy in systemic IF management. Previous reports have highlighted the potential clinical benefits of combining aTRB with voriconazole [9] or L-Amb [10] in IF. However, recovery of the underlying malignancies might be an important confounder to purported clinical benefit. Although the recent global guideline issued by the European Confederation of Medical Mycology in cooperation with the International Society for Human and Animal Mycology and the American Society for Microbiology [11] recommends consideration of the use of TRB in combination therapy for rare mold infections, this recommendation is based on several small series or registry data (with very few patients with hematological cancer) that were extremely heterogeneous in treatment scenarios and the confounding effect of surgery. In particular, data on the use of aTRB for treating disseminated IF in hematological cancer are very limited.

Altogether, our observations indicate that, although the combination of aTRB with systemic triazoles may be effective for localized skin infections or onychomycosis, there are concerns about the translatability of these benefits to systemic IF outcomes, especially in the setting of persistent immune deficits. Our findings are a reminder that, in addition to its in vitro activity, a drug's in vivo efficacy heavily depends on its pharmacokinetic behavior [12].

## Notes

***Financial support.*** This manuscript was supported by the Robert C. Hickey Chair in Clinical Care endowment, University of Texas MD Anderson Cancer Center (to D. P. K).
